# Effectiveness and Sustainability of an Antimicrobial Stewardship Program for Perioperative Prophylaxis in Pediatric Surgery

**DOI:** 10.3390/pathogens9060490

**Published:** 2020-06-19

**Authors:** Daniele Donà, Dora Luise, Elisa Barbieri, Nicola Masiero, Sonia Maita, Luca Antoniello, Theoklis Zaoutis, Carlo Giaquinto, Piergiorgio Gamba

**Affiliations:** 1 Paediatric Infectious Diseases Unit—Department of Women’s and Children’s Health, University of Padova, 35128 Padova, Italy; elisa.barbieri.5@phd.unipd.it (E.B.); nicola.masiero.5@studenti.unipd.it (N.M.); carlo.giaquinto@unipd.it (C.G.); 2 Infectious Diseases Department, Verona University Hospital, 37134 Verona, Italy; luise.dora@gmail.com; 3 Paediatric Surgery Unit—Department of Women’s and Children’s Health, University of Padova, 35128 Padova, Italy; sonia.maita@aopd.veneto.it (S.M.); lucamaria.antoniello@aopd.veneto.it (L.A.); piergiorgio.gamba@unipd.it (P.G.); 4 Division of Infectious Diseases, Department of Medicine, The Children’s Hospital of Philadelphia, Philadelphia, PA 19104, USA; ZAOUTIS@email.chop.edu

**Keywords:** antimicrobial stewardship, surgical site infection, perioperative prophylaxis, pediatric surgery, clinical pathways

## Abstract

Background—Appropriate perioperative antibiotic prophylaxis (PAP) is essential to prevent surgical site infections (SSIs) and to avoid antibiotics misuse. Aim—The aim of this study is to determine the effectiveness and long-term sustainability of an antimicrobial stewardship program (ASP), based on a clinical pathway (CP) and periodic education, to improve adherence to the guidelines for PAP in a tertiary care pediatric surgery center. Methods—We assessed the changes in PAP correctness and its effect on SSIs between the six months before and the 24 months after the implementation of ASP in the Pediatric Surgery Unit of the Department of Women’s and Children’s Health of Padova. The ASP was addressed to all surgeons and anesthesiologists of the Pediatric Surgery Unit. The primary outcome was appropriateness of PAP (agent, timing of the first dose, and duration). SSI rate was the secondary outcome. Results—1771 patients were included in the study and 676 received PAP. The overall correctness of the PAP, in terms of agent, timing, and duration, increased significantly after the CP implementation. What changed most was the PAP discontinuation within 24 h (*p* < 0.001). Cefazolin was the most used antibiotic, with a significant increase in the post-intervention period (*p* < 0.001) and with a reduction in the use of other broad-spectrum antibiotics. No variations in the incidence of SSIs were reported in the five periods (*p* = 0.958). Conclusion—The implementation of an ASP based on CP and education is an effective and sustainable antimicrobial stewardship tool for improving the correct use of PAP.

## 1. Introduction

Perioperative antibiotic prophylaxis (PAP) is essential to prevent surgical site infections (SSIs), which represent the second most common healthcare-associated infection and, according to the Centers for Diseases Control, complicate around 5% of surgical procedures every year [1,2].

Controlling and preventing antibiotic misuse through antimicrobial stewardship programs (ASPs), on the other hand, is crucial to prevent the incidence of bacterial resistances that are a well-known threat for hospitalized patients [3].

Most of the studies investigating ASPs for PAP are targeted to the adult population, with only few focusing on children and limited data on the efficacy and safety of these strategies [4,5,6,7]. For this reason, our study group conducted a first study in 2016–2017 with the aim of determining the effectiveness of an ASP based on a clinical pathway (CP) to improve the adherence to PAP guidelines in our Pediatric Surgery Unit [8]. The results showed a positive impact of the intervention, which was followed by an increase in appropriate PAP administration and in the selection of the appropriate antibiotic for prophylaxis, with a reduction in use of combination therapies and broad-spectrum antibiotics. The duration of prophylaxis decreased as well, and despite the greater use of narrow-spectrum antibiotic for fewer days, no increase in treatment failures and SSIs rate was reported.

This current work represents a re-elaboration and extension of that first analysis, with a period of observations that stretches to 2019, including 24 months after the first implementation of the ASP, in order to determine the long-term effectiveness and sustainability of the intervention.

## 2. Methods

### 2.1. Design and Timing

We assessed the changes in PAP correctness and its effect on SSIs in the 24 months after the implementation of ASP in the Pediatric Surgery Unit of the Department of Women’s and Children’s Health of Padova, a third-level university hospital that represents the referral center for pediatric surgery for the whole Veneto region (population: 4905 million people).

**Figure 1:**
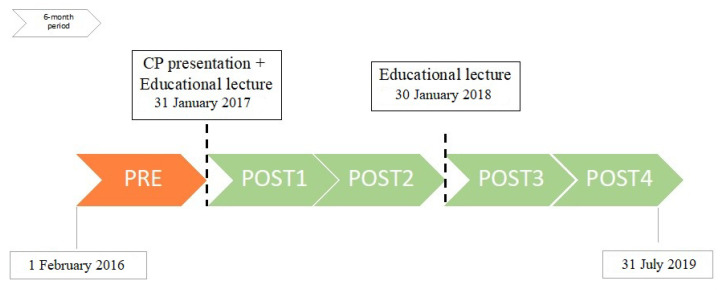
Study timeline with interventions.

The 30 month study period was divided into the pre-intervention period (PRE, February 1st–July 31st 2016) and four post-intervention periods (POST 1: February 1st–July 1st 2017; POST 2: August 1st, 2017–January 31st, 2018; POST 3: 1 February–31 July 2018; POST 4: 1 August 2018–31 January 2019). The study timeline is shown in Figure 1.

### 2.2. Population

We selected and analyzed data from the medical records of all the pediatric patients (age 1 month–18 years) hospitalized in the department who underwent a surgical procedure in the study period.

Exclusion criteria were as follows:

- Patients with concurrent diagnosis of infections at the moment of surgical operation, or receiving antimicrobial therapy for more than 24 h at the moment of surgical operation;
- Patients receiving immunosuppressive therapy, or with pre-existing immunodeficiencies;
- Patients hospitalized for neurosurgery, cardiac surgery, vascular surgery, ENT surgery, ophthalmic surgery, injections of sclerotizing drugs, positioning of central lines, and removal of gastrostomies.

### 2.3. Intervention

The antimicrobial stewardship intervention consisted of a lecture held by the pediatric infectious diseases specialists and directed to all the medical staff of the Pediatric Surgery Department that took place on January 31 2017. The topics of the lecture were as follows:

- overview of epidemiology and microbiology of SSIs;
- the ASHP-IDSA-SHEA-SIS 2013 Guidelines for PAP [9];
- benefits of correct antimicrobial prescription;
- rates of antimicrobial resistances in Padua Hospital;
- data from the PRE intervention period and potential areas of improvement;
- peculiar practices of the Pediatric Surgery Unit and questions from the medical staff.

At the end of the lecture, clinical pathways (CPs) were presented in the form of pocket cards detailing all the steps needed to administer correct PAP. These pocket cards were given to every medical doctor.

CPs were developed by a multidisciplinary group (pediatric infectious disease, microbiology, and pediatric surgery) based on the aforementioned guidelines [9], considering our local microbiology data, and with the supervision of the pediatric infectious diseases team of Philadelphia Children’s Hospital.

For a detailed description of the CPs, complete with pocket card images, see the previous study [8].

A second lecture was held after one year from the implementation of CPs, on January 30 2018. This second meeting included all the main topics of the first one, as well as preliminary post-implementation data.

### 2.4. Outcomes and Variables

The following information was recorded for every patient:

(1) demographic and preoperative data including sex, age, weight;
(2) perioperative data including type of procedure (divided for major categories, according to the International Classification of Diseases, 9th revision and Clinical Modification (ICD-9-CM), wound class (divided in clean, clean-contaminated, contaminated, and dirty/infected, according to the CDC’s classification [10]), operative time, urgency of procedure, and length of hospital stay;
(3) perioperative PAP data, such as indication for PAP, administration of PAP, and, among those who received PAP, correctness of both antimicrobial agent and time of antibiotic discontinuation (as defined by the guidelines).
(4) post-procedure data including occurrence of any SSI.

The main outcome was the appropriateness of PAP, defined as the administration of the correct antimicrobial agent for the specific surgical procedures with the correct timing of administration and correct discontinuation within 24 postoperative hours, according to the clinical practice guidelines for antimicrobial prophylaxis in surgery [9].

The secondary outcome was the incidence of SSIs within 6 months after the surgical procedure.

### 2.5. Data Collection and Statistical Analysis

Data were collected manually from electronic medical records (Galileo system), and paper medical records, including all documents published within 6 months after the patients’ discharge, in order to identify the incidence of SSIs. We used password-protected REDCap^®^ data collection forms, and we stored them in the secure server at the University of Padua. Patient’s demographic and clinical data were analyzed in a descriptive way: continuous variables have been described with mean, median, and minimum/maximum values, while discrete variables have been described with frequencies and percentages.

Comparison of categorical independent data was performed with chi-square test. In the comparison of continuous, parametric, and normally distributed variables Fisher and Welch one-way ANOVA tests were used, while for continuous, non-parametric variables, Kruskal–Wallis rank sum test was performed.

Data were analyzed with R 3.6 statistical software (R Foundation for Statistical Computing, Vienna, Austria) for Windows. Statistical significance was confirmed with *p* < 0.05.

### 2.6. Privacy and Ethical Aspects

Privacy was guaranteed in two ways: a unique, study-specific survey number was assigned to each patient and no personally identifying data were collected. This study was approved by the Research Ethics Committee of the Department of Women’s and Children’s Health of Padova.

## 3. Results

### 3.1. Patient Demographics and Procedure Characteristics

During the study period, 1771 patients with a median age of 5.2 years (0–17) were included, their demographic characteristics, as well as complete data regarding wound class, type of surgical procedure, and procedure urgency are displayed in Table 1.

Most of the wounds were classified as clean: 74.7% patients had a clean wound in the PRE period vs 74.4%, 79.9%, 75.4%, and 73.5% in the POST periods. Around 10% of the wounds in the PRE and POST2, POST3, and POST4 were classified as contaminated and no wound was dirty or infected. 

The most represented types of surgical procedure were umbilical hernia/inguinal hernia and scrotum surgery, with no difference between the PRE period (38.8%) and POST periods (37.1% in POST1, 40.4% in POST2, 39.0% in POST3, and 37.8% in POST4). The second most frequent type of surgical procedure was head/neck surgery.

Considering surgical procedure urgency, 9.4% were classified as urgent with no significant difference between periods.

### 3.2. Perioperative Antibiotic Prophylaxis

Table 2 shows complete data regarding changes in PAP in the different periods.

In the PRE period, PAP was administered in 161/371 (43.4%) procedures, in the POST1 in 134/353 (38.0%), in the POST2 in 122/363 (34.3%), in the POST3 in 127/366 (34.7%), and in the POST4 in 132/325 (40.6%).

In more than 80% of cases, PAP was administered with a correct indication (131/161 (81.4%) in PRE vs. 115/134 (85.9%) in POST1, 105/122 (86.0%) in POST2, 112/127 (88.2%) in POST3, and 119/132 (90.2%) in POST4) and with a correct timing of first dose administration. 

After CP implementation, the frequency of PAP discontinuation within 24 h was significantly higher than before, varying from 68/161 (42.2%) in the PRE period to 109/132 (82.6%) in the POST4 period (*p* < 0.001).

Considering overall PAP correctness, in the PRE period just 65/161 (40.4%) PAPs were correct, increasing significantly in the POST period (*p* < 0.001) with a peak of 93/127 (73.2%) correct PAPs in POST3 period.

**Figure 2:**
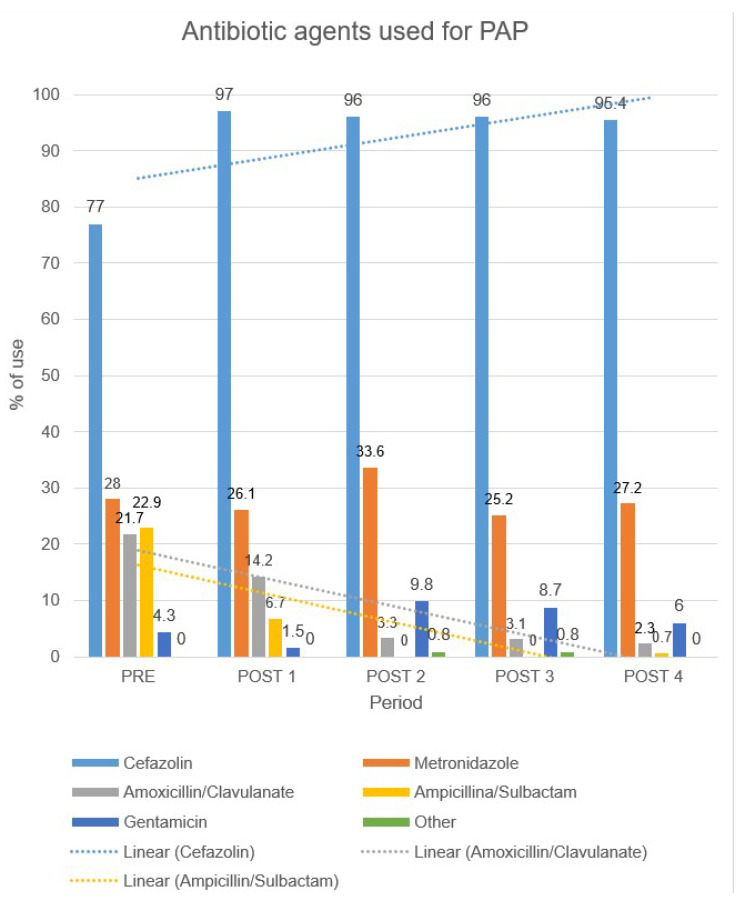
Antibiotic agents used for perioperative antibiotic prophylaxis (PAP).

The most used antibiotic for PAP was cefazolin, which increased significantly after CP implementation, from 77.0 in PRE to 97.0% in POST1 (*p* < 0.001) and steadily remained >95% of use (96.0% in POST2, 96.0% in POST3, and 95.4% in POST4). The second most used antibiotic was metronidazole, followed by amoxicillin and clavulanic acid, which decreased significantly after CP implementation, from 21.7% in PRE to 2.3% in POST4, (*p* < 0.001). Ampicillin–sulbactam, which was used in 22.9% of cases during the PRE period, almost disappeared after implementation of CP, while gentamicin frequency of use was not affected by CP implementation (*p* = 0.120). Antibiotic use is displayed in Figure 2.

Monotherapy was adopted in around 70% of PAP administrations with no significant differences in the various periods.

### 3.3. Surgical Site Infections (SSIs)

SSI rate did not change significantly in the different periods: 10/371 (2.7%) SSIs were reported in PRE, 7/353 (2.0%) in POST1, 8/356 (2.2%) in POST2, 7/366 (1.9%) in POST3, and 7/325 (2.2%) in POST4; *p* = 0.958.

## 4. Discussion

Perioperative antibiotic prophylaxis is the most efficacious way to prevent SSIs [1]. 

The most recent guidelines defining PAP recommends narrow-spectrum antibiotics with a duration of less than 24 h as first choice for all surgical operations, with the exception of cardiac surgery.

A recently published systematic scoping review on antibiotic stewardship in pediatrics [11] reported that so far only eight studies focused on PAP. Six showing an improvement in antibiotic usage appropriateness after PAP guideline implementation [4,5,6,12,13,14], one not reporting any change in PAP adherence after a checklist and antibiotic order list implementation [7], and one [15] reporting no significant changes in hospital-associated infection rate nor in multidrug-resistant organism rate.

Our previously published article on CP’s impact on PAP guideline adherence confirmed other research findings with a significantly increased rate in narrow-spectrum antibiotic usage [8]. 

One interesting result of this study is that the rate of PAP correctly discontinued within 24 h after surgery increased significantly (*p* < 0.001) after CP introduction varying from 42.2% in the PRE period to 82.6% in the POST4 period. It is worth noting that the rate of correctly discontinued PAP increased more in the POST3 (89.0%) and POST4 period, after the second educational-feedback lecture. There are still uncertainties in stopping the PAP after 24 h especially in more complex or prolonged surgeries [5,16] or with the presence of a urinary catheter [8], but our results show no difference in SSI rate even with higher rates of early discontinuation.

As regards the antimicrobial agents used for PAP, cefazolin was the most used antibiotic in all the periods and there was a statistically significant variation after CP implementation (*p* < 0.001). According to PAP CP, cefazolin represents the first-choice antibiotic in all surgical procedures because of its narrow-spectrum focus especially versus *Staphylococcus aureus, Coagulase-negative staphylococci* and Gram-negative bacteria as well as its low cost.

Metronidazole is used in combination with cefazolin in gastro-intestinal surgery such as gastric or intestinal obstruction, appendicitis, and bile duct procedures with possible manipulation. In case the patient is allergic to beta-lactams, the antibiotic, ciprofloxacin is used instead of cefazolin for the same procedures.

Amoxicillin and clavulanic acid as well as ampicillin and sulbactam rates were statistically reduced in the different periods (*p* < 0.001). These protected-penicillins represent a good indicator of PAP guidelines adherence.

Lastly, gentamicin rate did not decrease after PAP CP implementation: this antibiotic should be used in combination with clindamycin in patients who are allergic to penicillins and/or cephalosporins in gastro-intestinal procedures involving the upper and lower digestive tract except the intestinum crassum, in laparoscopy procedures involving bile duct or urinary tract, and when head/neck surgery wound are classified as clean-contaminated. Indeed, just one patient was allergic to penicillins/cephalosporins during the entire study period, thus the use of gentamicin was not supported in the other 31 cases. Furthermore, gentamicin was never administered alone and was most frequently used in combination with cefazolin and metronidazole, and regarding monotherapy versus combinations, there was no difference between different periods as well (*p* = 0.332).

A possible explanation of non-correct PAP administration, considering (either) both timing of the first dose (or) either antibiotic type, could be that pediatric PAP guidelines were developed deriving data from adult patients: this could lead to the misconception that these guidelines are not relevant for pediatric patients. A possible solution to this problem could be having a pediatric surgeon as part of the antibiotic stewardship team focusing on audit and feedback [5] as well as a pharmacist focusing on optimizing the antibiotics order process [7].

Lastly, non-adherence to PAP CP could be overcome by increasing evidence-based knowledge on this matter.

As a matter of facts, SSI rate was used to control PAP guideline appropriateness on local epidemiology and there was no statistical difference in SSI rate before and after CP introduction. With a mean of 2% SSI rate, our results were in line with previous findings reporting 5% of SSI rate yearly [2,8], thus proving the safety of the intervention. 

### Strengths and Limitations

This is the first study that evaluates the long-term effectiveness and sustainability in an Italian university hospital. This intervention has been developed by a multidisciplinary team constituted by infectious diseases pediatricians, pediatric surgeons, and microbiologists in order to be adaptable to other settings in other hospitals and it proved to be feasible and low cost.

Another strength of this study is the long follow-up period (24 months) that is essential to study antibiotic stewardship intervention sustainability in time, especially with the aim of adapting the tool to be more practical for medical doctors.

On the other hand, the retrospective design and the patients’ follow-up limited to our center represent a limitation. In addition, our study is only limited to specific surgical operations, as nose–throat surgical procedures as well as cardio surgery and neurosurgery were not included in the analysis. 

## 5. Conclusions

The implementation of a CP, supported by periodic educational interventions, proved to be a valid means of antimicrobial stewardship in the Pediatric Surgery Unit. The increase of the correct indication, the antibiotic discontinuation within the twenty-four hours, and the use of first-line antibiotics with the reduction of broad-spectrum ones configures it as an effective and sustainable tool for improving the correct use of PAP.

## Figures and Tables

**Table 1:** Patient demographic and procedure characteristics.

Patients’ Characteristics	PRE *n* = 371	POST 1 *n* = 353	POST 2 *n* = 356	POST 3 *n* = 366	POST 4 *n* = 325
**Sex** Male Female	- 256 (69.0%) 115 (31.0%)	- 260 (73.6%) 93 (26.4%)	- 245 (68.8%) 111 (31.2%)	- 253 (69.1%) 113 (31.4%)	- 223 (68.6%) 102 (31.4%)
**Age (median, years)**	5.2 (0–17)	5.0 (0–17)	5.5 (0–17)	6.1 (0–17)	5.5 (0–17)
**Median weight (kg)**	20.0 (2.3–74.0)	19.0 (2.1–72.0)	20.0 (2.6–84.5)	20.0 (2.3–101.0)	20.0 (3.1–61.0)
Wound class (*) Clean Clean-contaminated Contaminated Dirty	- 277 (74.7%) 63 (17%) 31 (8.3%) 0 (0.0%)	- 282 (79.9%) 52 (14.7%) 19 (5.4%) 0 (0.0%)	- 242 (68%) 76 (21.3%) 38 (10.7%) 0 (0.0%)	- 276 (75.7%) 60 (16.4%) 30 (8.2%) 0 (0.0%)	- 239 (73.5%) 51 (15.7%) 35 (10.8%) 0 (0.0%)
**Type of surgical operation** Appendectomy Gastrointestinal tract/liver/biliary tract Head/neck Umbilical/inguinal/scrotum Skin/soft tissue Pediatric gynecology Chest Urinary tract Other	- 24 (6.5%) 42 (11.3%) 53 (14.3%) 144 (38.8%) 44 (11.9%) 6 (1.6%) 16 (4.3%) 17 (4.6%) 25 (6.7%)	- 21 (5.9%) 31 (8.8%) 71 (20.1%) 131 (37.1%) 30 (8.5%) 9 (2.5%) 28 (7.9%) 12 (3.4%) 20 (5.8%)	- 36 (10.1%) 46 (12.9%) 42 (11.8%) 144 (40.4%) 41 (11.5%) 4 (1.1%) 18 (5.1%) 9 (2.5%) 16 (4.6%)	- 24 (6.6%) 51 (13.9%) 46 (12.6%) 143 (39.0%) 44 (12.0%) 5 (1.4%) 16 (4.4%) 9 (2.5%) 28 (7.6%)	- 29 (8.9%) 41 (12.6%) 43 (13.2%) 123 (37.8%) 35 (10.8%) 4 (1.2%) 21 (6.5%) 7 (2.2%) 22 (6.8%)
**Urgent procedure** Yes No	- 24 (6.5%) 347 (93.5%)	- 38 (10.8%) 315 (89.2%)	- 38 (10.7%) 318 (89.3%)	- 29 (10.6%) 327 (89.4%)	- 28 (8.7%) 297 (91.3%)
**Administration of PAP** Yes No	- 161 (43.4%) 210 (56.6%)	- 134 (38.0%) 219 (62.0%)	- 122 (34.3%) 234 (65.7%)	- 127 (34.7%) 239 (65.3%)	- 132 (40.6%) 193 (59.4%)

* Wound class was classified according to the CDC’s classification [10].

**Table 2:** Perioperative antibiotic prophylaxis (PAP) characteristics.

PAP’s Characteristics	PRE *n* = 161	POST 1 *n* = 134	POST 2 *n* = 122	POST 3 *n* = 127	POST 4 *n* = 132	*p* Value
**Correct indication** Yes No	- 131 (81.4%) 30 (18.6%)	- 115 (85.9%) 19 (14.1%)	- 105 (86.0%) 17 (14.0%)	- 112 (88.2%) 15 (11.8%)	- 119 (90.2%) 13 (9.8%)	- 0.259
**Correct timing of first dose** Yes No	- - 138 (85.7%) 23 (14.3%)	- - 118 (88.0%) 16 (12.0%)	- - 102 (83.6%) 20 (16.4%)	- - 109 (85.8%) 18 (14.2%)	- - 112 (84.8%) 20 (15.1%)	- - 0.891
**Discontinuation within 24 h** Yes No	- - 68 (42.2%) 93 (57.8%)	- - 90 (67.2%) 44 (32.1%)	- - 95 (77.9%) 27 (22.1%)	- - 113 (89.0%) 14 (11.0%)	- - 109 (82.6%) 23 (17.4%)	- - <0.001
**Overall correct PAP** Yes No	- 65 (40.4%) 96 (59.6%)	- 81 (60.4%) 53 (39.6%)	- 77 (63.1%) 45 (36.9%)	- 93 (73.2%) 34 (26.8%)	- 88 (66.7%) 44 (33.3%)	- <0.001
**Antibiotic agent** Cefazolin Metronidazole Amoxi/clav Ampi/sulb Gentamicin Other	- 124 (77.0%) 45 (28.0%) 35 (21.7%) 37 (22.9%) 7 (4.3%) 0 (0.0%)	- 130 (97.0%) 35 (26.1%) 19 (14.2%) 9 (6.7%) 5 (1.5%) 1 (0.7%)	- 117 (96.0%) 41 (33.6%) 4 (3.3%) 0 (0.0%) 12 (9.8%) 1 (0.8%)	- 122 (96.0%) 32 (25.2%) 4 (3.1%) 0 (0.0%) 11 (8.7%) 1 (0.8%)	- 126 (95.4%) 36 (27.2%) 3 (2.3%) 1 (0.7%) 8 (6.0%) 0 (0.0%)	- <0.001 0.614 <0.001 <0.001 0.120 0.670
**Monotherapy** **Combinations**	114 (70.8%) 47 (29.2%)	105 (78.4%) 29 (21.6%)	82 (67.2%) 40 (32.8%)	95 (74.8%) 32 (25.2%)	96 (72.7%) 36 (27.3%)	0.332

## Abbreviations

PAP	Perioperative antibiotic prophylaxis
CP	Clinical pathway
ASP	Antibiotic stewardship program
SSI	Surgical site infection
ID	Infectious disease

## References

[1] Magill S.S., Edwards J.R., Bamberg W., Beldavs Z.G., Dumyati G., Kainer M.A., Lynfield R., Maloney M., McAllister-Hollod L., Nadle J. (2014). Multistate point-prevalence survey of health care-associated infections. N. Engl. J. Med..

[2] Weigelt J.A., Lipsky B.A., Tabak Y.P., Derby K.G., Kim M., Gupta V. (2010). Surgical site infections: Causative pathogens and associated outcomes. Am. J. Infect. Control.

[3] Llor C., Bjerrum L. (2014). Antimicrobial resistance: Risk associated with antibiotic overuse and initiatives to reduce the problem. Ther. Adv. Drug Saf..

[4] Caruso T.J., Wang E., Schwenk H.T., Scheinker D., Yeverino C., Tweedy M., Maheru M., Sharek P.J. (2017). A quality improvement initiative to optimize dosing of surgical antimicrobial prophylaxis. Pediatr. Anesth..

[5] Dimopoulou A., Kourlaba G., Psarris A., Coffin S., Spoulou V., Zaoutis T. (2016). Perioperative antimicrobial prophylaxis in pediatric patients in Greece: Compliance with guidelines and impact of an educational intervention. J. Pediatr. Surg..

[6] So J.P., Aleem I.S., Tsang D.S., Matlow A.G., Wright J.G., Surgical Site Infection Task Force (2015). Increasing Compliance with an Antibiotic Prophylaxis Guideline to Prevent Pediatric Surgical Site Infection. Ann. Surg..

[7] Putnam L.R., Chang C.M., Rogers N.B., Podolnick J.M., Sakhuja S., Matusczcak M. (2015). Adherence to surgical antibiotic prophylaxis remains a challenge despite multifaceted interventions. Surgery.

[8] Donà D., Luise D., La Pergola E., Montemezzo G., Frigo A., Lundin R., Zaoutis T., Gamba P., Giaquinto C. (2019). Effects of an antimicrobial stewardship intervention on perioperative antibiotic prophylaxis in pediatrics. Antimicrob. Resist. Infect. Control.

[9] Bratzler D.W., Dellinger E.P., Olsen K.M., Perl T.M., Auwaerter P.G., Bolon M.K., Fish D.N., Napolitano L.M., Sawyer R.G., Slain D. (2013). Clinical practice guidelines for antimicrobial prophylaxis in surgery. Surg. Infect..

[10] Devaney L., Rowell K.S. (2004). Improving surgical wound classification—Why it matters. AORN J..

[11] Donà D., Barbieri E., Daverio M., Lundin R., Giaquinto C., Zaoutis T., Sharland M. (2020). Implementation and impact of pediatric antimicrobial stewardship programs: A systematic scoping review. Antimicrob. Resist. Infect. Control.

[12] Degli Atti M.C., Alegiani S.S., Raschetti R., Arace P., Giusti A., Spiazzi R., Raponi M. (2017). A collaborative intervention to improve surgical antibiotic prophylaxis in children: Results from a prospective multicenter study. Eur. J. Clin. Pharmacol..

[13] Huebner J., Rack-Hoch A., Pecar A., Schmid I., Klein C., Borde J. (2013). Pilotprojekt einer pädiatrischen Antibiotic-Stewardship-Initiative am Dr. von Haunerschen Kinderspital-neue Wege der pädiatrischen Infektiologie. Klinische Pädiatrie.

[14] Ruvinskya S., Mónacoa A., Péreza G., Taicza M., Indaa L., Epelbauma C. (2014). Effectiveness of a program to improve antibiotic use in children hospitalized in a children’s tertiary care facility in Argentina. Arch. Argent. Pediatr..

[15] Walker S., Datta A., Massoumi R.L., Gross E.R., Uhing M., Arca M.J. (2017). Antibiotic stewardship in the newborn surgical patient: A quality improvement project in the neonatal intensive care unit. Surgery.

[16] Rangel S.J., Fung M., Graham D.A., Ma L., Nelson C.P., Sandora T.J. (2011). Recent trends in the use of antibiotic prophylaxis in pediatric surgery. J. Pediatr. Surg..

